# PHA4GE quality control contextual data tags: standardized annotations for sharing public health sequence datasets with known quality issues to facilitate testing and training

**DOI:** 10.1099/mgen.0.001260

**Published:** 2024-06-11

**Authors:** Emma J. Griffiths, Inês Mendes, Finlay Maguire, Jennifer L. Guthrie, Bryan A. Wee, Sarah Schmedes, Kathryn Holt, Chanchal Yadav, Rhiannon Cameron, Charlotte Barclay, Damion Dooley, Duncan MacCannell, Leonid Chindelevitch, Ilene Karsch-Mizrachi, Zahra Waheed, Lee Katz, Robert Petit III, Mugdha Dave, Paul Oluniyi, Muhammad Ibtisam Nasar, Amogelang Raphenya, William W. L. Hsiao, Ruth E. Timme

**Affiliations:** 1 Centre for Infectious Disease Genomics and One Health, Faculty of Health Sciences, Simon Fraser University, Burnaby, British Columbia, Canada; 2 Theiagen Genomics, LLC, Highlands Ranch, Colorado, USALLC, Highlands Ranch, Colorado, USA; 3 Department of Community Health & Epidemiology, Faculty of Medicine, Dalhousie University, Halifax, Nova Scotia, Canada, and Faculty of Computer Science, Dalhousie University, Halifax, Nova Scotia, Canada; 4 Department of Microbiology & Immunology, Western University, London, Ontario, Canada; 5 The Roslin Institute, University of Edinburgh, Edinburgh, UK; 6 National Center for Emerging and Zoonotic Infectious Diseases, Centers for Disease Control and Prevention, Georgia, USA; 7 National Microbiology Laboratory, Public health Agency of Canada, Winnipeg, MB, Canada; 8 Department of Infection Biology, London School of Hygiene and Tropical Medicine, London, UK; 9 MRC Centre for Global Infectious Disease Analysis, School of Public Health, Imperial College London, London, UK; 10 National Center for Biotechnology Information, National Library of Medicine, National Institutes of Health, Bethesda, MD, USA; 11 European Bioinformatics Institute, Wellcome Genome Campus, Hinxton, UK; 12 Center for Food Safety, University of Georgia, Georgia, USA; 13 Wyoming Public Health Laboratory, Wyoming, USA; 14 McMaster University, Hamilton, Ontario, Canada; 15 Chan Zuckerberg Biohub, San Francisco, CA, USA; 16 Department of Biology, College of Science, United Arab Emirates University- AL Ain, Abu Dhabi, UAE; 17 Michael G. DeGroote Institute for Infectious Disease Research, McMaster University, Hamilton, Ontario, Canada; 18 Center for Food Safety and Applied Nutrition, U.S. Food and Drug Administration, College Park, Maryland, USA

**Keywords:** contextual data, data sharing, public health genomics, quality control, reference datasets

## Abstract

As public health laboratories expand their genomic sequencing and bioinformatics capacity for the surveillance of different pathogens, labs must carry out robust validation, training, and optimization of wet- and dry-lab procedures. Achieving these goals for algorithms, pipelines and instruments often requires that lower quality datasets be made available for analysis and comparison alongside those of higher quality. This range of data quality in reference sets can complicate the sharing of sub-optimal datasets that are vital for the community and for the reproducibility of assays. Sharing of useful, but sub-optimal datasets requires careful annotation and documentation of known issues to enable appropriate interpretation, avoid being mistaken for better quality information, and for these data (and their derivatives) to be easily identifiable in repositories. Unfortunately, there are currently no standardized attributes or mechanisms for tagging poor-quality datasets, or datasets generated for a specific purpose, to maximize their utility, searchability, accessibility and reuse. The Public Health Alliance for Genomic Epidemiology (PHA4GE) is an international community of scientists from public health, industry and academia focused on improving the reproducibility, interoperability, portability, and openness of public health bioinformatic software, skills, tools and data. To address the challenges of sharing lower quality datasets, PHA4GE has developed a set of standardized contextual data tags, namely fields and terms, that can be included in public repository submissions as a means of flagging pathogen sequence data with known quality issues, increasing their discoverability. The contextual data tags were developed through consultations with the community including input from the International Nucleotide Sequence Data Collaboration (INSDC), and have been standardized using ontologies - community-based resources for defining the tag properties and the relationships between them. The standardized tags are agnostic to the organism and the sequencing technique used and thus can be applied to data generated from any pathogen using an array of sequencing techniques. The tags can also be applied to synthetic (lab created) data. The list of standardized tags is maintained by PHA4GE and can be found at https://github.com/pha4ge/contextual_data_QC_tags. Definitions, ontology IDs, examples of use, as well as a JSON representation, are provided. The PHA4GE QC tags were tested, and are now implemented, by the FDA’s GenomeTrakr laboratory network as part of its routine submission process for SARS-CoV-2 wastewater surveillance. We hope that these simple, standardized tags will help improve communication regarding quality control in public repositories, in addition to making datasets of variable quality more easily identifiable. Suggestions for additional tags can be submitted to PHA4GE via the New Term Request Form in the GitHub repository. By providing a mechanism for feedback and suggestions, we also expect that the tags will evolve with the needs of the community.

**Significance as a BioResource to the community**

Before public health genomic datasets can be re-used for different applications, they must be assessed against pre-defined quality criteria and methods. Public health surveillance programs often implement strict QC thresholds, and if generated data does not meet those thresholds labs may not feel like it is appropriate (or may not have permission) to share data. However, it is important to recognize that data that is considered insufficient for one purpose may be appropriate and usable for other public health applications. While quality assessment annotations in contextual data records can enable data users to quickly triage data for its utility and enable the labelling and identification of datasets with known issues useful for training of software and personnel, there is currently no such set of standardized annotations. In response to this need, the Public Health Alliance for Genomic Epidemiology (PHA4GE) has created five standardized fields (‘tags’) as well as associated picklists, for inclusion in public and private records to facilitate searches and quality assessments. In doing so, we hope the tags empower data generators to share a greater variety of data to support a wider array of public health analyses, as well as the optimization of protocols and processes.

## Data Summary

The software used in this study is available on GitHub.

Project name: PHA4GE QC Contextual Data Tags Specification

Project home page: https://github.com/pha4ge/contextual_data_QC_tags

Operating system: Platform independent

Programming language: Not applicable

Other requirements: None

License: MIT License

## Introduction

Pathogen genomics surveillance laboratories generate microbial sequence data that can be used in a variety of ways. Examples include the detection and resolution of outbreaks, development of vaccines and diagnostic tests, understanding microbial evolution including antimicrobial resistance and virulence mechanisms, detection of zoonotic events and patterns of transmission, source attribution, and more [1,7]. The quality of sequence datasets greatly impacts their utility, the interpretations of analytical results, and the decisions that can be made based on how much confidence one has in the interpretations [8,9]. How data quality is evaluated depends on defined criteria, metrics and thresholds – which may vary depending on the pathogen, use case, and initiative. While guidance exists for certain types of data quality assessments, there are also a wide range of opinions and methods, meaning that the definition of ‘higher quality’ or ‘lower quality’ data depends on the laboratory generating or using the data. Simply put, ‘lower quality’ data is any data that does meet a particular set of quality assessment criteria. The quality of sequence data can vary for many reasons, including, but not limited to, low concentrations of starting materials, expired reagents, deviations from ideal sample handling and storage conditions, errors during library preparation such as overloading or in the case of long-read sequencing techniques underloading of flow cells, and contamination within and between sequencing runs [10,11]. Quality control metrics of raw reads depend on many factors such as the depth and breadth of coverage of generated reads compared to a reference, the presence of reads from another source (i.e. previous run, host organism, sample contamination) and the number and density of flow cell clusters in a run [12]. Quality control metrics and thresholds often differ across laboratories and surveillance networks. While public health laboratories generate and release a vast number of high quality sequences, there will often be a proportion of datasets that may fall just short of a set of prescribed baseline quality control metrics. These datasets are then excluded from many types of public health analyses and are often not publicly released. In these cases, the issues associated with these borderline or lower quality datasets have often been identified, and the datasets can still provide important surveillance insights or information on real-world test performance. Conversely, lower quality datasets can sometimes be included in public repository submissions but are not flagged which creates issues for laboratories using the data.

**Fig. 1.**
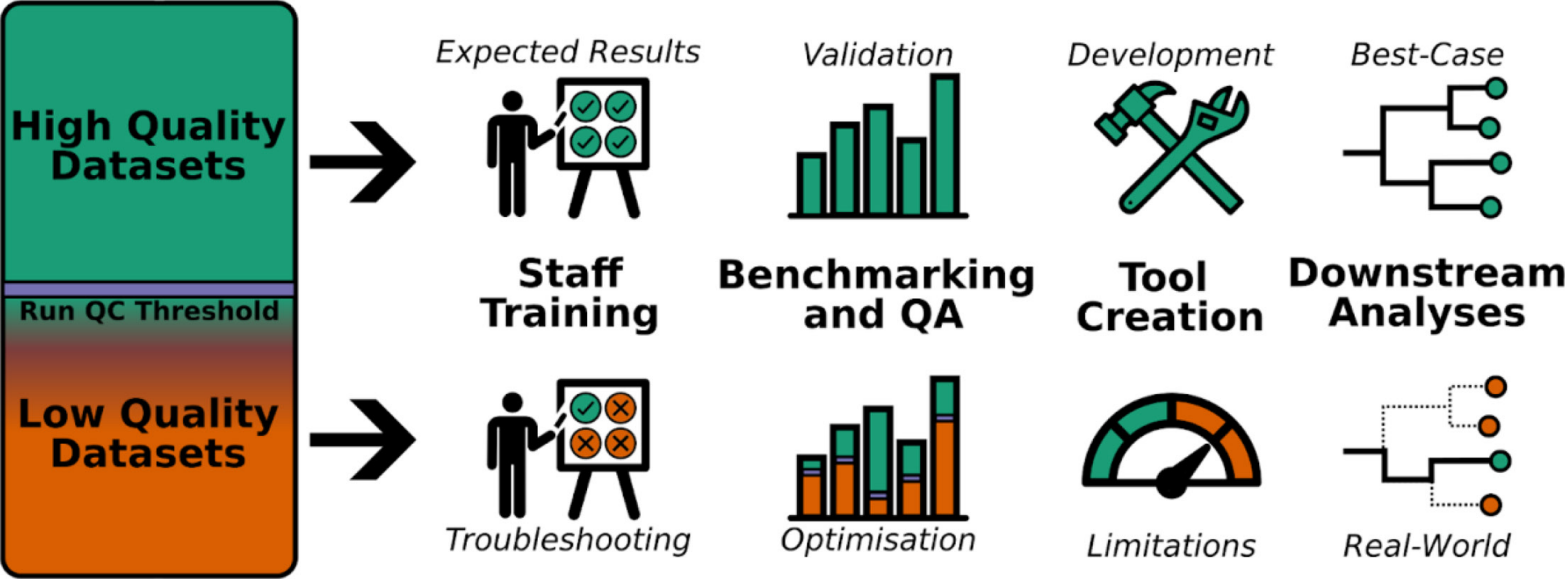
Sequence data quality is assessed using prescribed criteria (i.e. metrics) and thresholds. Datasets of high and lower quality have many uses in public health activities such as staff training and lab procedure/software optimization and validation in ideal and real-world scenarios.

As pathogen sequencing is increasingly used routinely in public health laboratories for the surveillance of different pathogens, and as programmes are continually developed and expanded, laboratories must carry out robust optimization of wet- and dry-lab procedures [13]. Lower quality datasets are highly useful for optimizing, validating, verifying, and benchmarking the performance of algorithms, pipelines and instruments, as well as training new personnel (Fig. 1). An example of the utility of high- and lower-quality datasets can be seen in Xiaoli *et al.* [14] in which SARS-CoV-2 Nanopore/Illumina read datasets generated from public health genomic surveillance were shared as a collection to support benchmarking tools, understanding the genomic epidemiology of different lineages, and identifying variants of concern. The collection also contained a number of SARS-CoV-2 genomes of lower quality due to recognized errors and common sequencing failures [14]. In addition to lower quality data, synthetic data – artificially created lab constructs as opposed to data generated from real-world samples – can be very useful for wet-lab testing, bioinformatic analysis, and workflow optimization, but is rarely shared and is often poorly annotated.

Sharing sub-optimal data can be useful for the broader public health and research community, particularly when the data are carefully annotated with known issues so that it is not mistaken for better quality information and can be more easily identified in repositories. However, there are currently no standardized attributes for tagging poor-quality datasets, preventing them from being easily searched and made accessible, and all but ensuring that they are excluded from applications that require only higher quality data. Standardized fields and terms have previously proven useful in improving data harmonization and integration as well as communication and data sharing in SARS-CoV-2 surveillance [15,16], and the implementation of genomics contextual data (metadata) standards have been encouraged repeatedly in the community [17,22]. As such, a set of standardized attributes to describe the properties, quality and purpose of microbial sequence datasets could make quality control results more explicit in public (and private) repositories.

The Public Health Alliance for Genomic Epidemiology (PHA4GE) is a global coalition of scientists focused on improving the reproducibility, interoperability, portability, and openness of public health bioinformatics software, data and expertise (https://pha4ge.org/). As part of its mission to improve interoperability and reproducibility, PHA4GE workgroups develop, share and promote consensus data specifications, in an effort to streamline and improve data structures across public health bioinformatics resources (tools, protocols, databases, platforms, and repositories). The overarching goal of this work is the development of an open software philosophy and ecosystem that will empower more stakeholders across global public health to analyse, manage and govern their own data, regardless of resource status. The Data Structures Working Group, tasked with assessing needs and developing these specifications, operates by consensus and comprises diverse perspectives and expertise with members representing many different countries, organisations, microbial sequencing initiatives, and standards development efforts.

To address the challenges of sharing lower quality datasets, PHA4GE has developed a set of standardized contextual data attributes (fields and terms known as ‘tags’) that can be included in public repository submissions as a means of flagging pathogen sequence data with known quality issues to increase their discoverability, and to facilitate their interpretation and appropriate reuse. The contextual data tags (attributes) were developed through a series of consultations with the public health microbiology research community, including input from the International Nucleotide Sequence Data Collaboration (INSDC), and staff from multiple national, regional and local public health institutions. The development of these tags, standardized using community-based resources known as ontologies, is expected to be an iterative and participatory process with input from users and subject matter experts from across the community. Ontologies are well-defined controlled vocabulary describing a domain, structured in a hierarchy where logical relationships link the terms, and the meanings of terms are disambiguated using persistent identifiers [23]. As ontologies are developed by community consensus, by applying ontology-based attributes in publicly available data, the pitfalls and variability of institution-specific vocabulary and free text can be avoided. The standardized tags are agnostic to the organism and sequencing technique used and can be applied to data generated from any pathogen using an array of sequencing techniques – enabling adoption of the tags across sequencing projects and platforms. The list of standardized tags for quality control, introduced here, is maintained by PHA4GE and can be found at https://github.com/pha4ge/contextual_data_QC_tags. Recognizing that data needs can change over time, or that different use cases can require additional vocabulary, PHA4GE accepts suggestions for new tags from the community which can be submitted using the New Term Request System on GitHub (see PHA4GE repository linked above).

PHA4GE’s quality control (QC) tags were piloted by the FDA’s GenomeTrakr network [24,25] during its pandemic expansion into metagenomic wastewater surveillance for COVID-19 [26], enabling transparent sharing of early-stage data at the US National Centre for Biotechnology Information (NCBI). Many datasets, especially early on, were flagged as lower quality with these tags, allowing a federal agency to comfortably share pandemic data while clearly communicating confidence levels. This practice of sharing data across the quality spectrum aids laboratories in method development and supports the biotech industry in refining diagnostic kits, particularly for challenging applications like sequencing SARS-CoV-2 from wastewater. The incorporation of these QC tags into GenomeTrakr’s standard submission procedures showcases their utility in enhancing data-sharing capabilities within pathogen genomic surveillance.

The sharing of lower quality datasets and their annotation using the contextual data tags described here will enable public health labs to make use of data that would have otherwise been discarded, and in some cases, side-step the need to generate synthetic data for representing different real-world scenarios. Using these tags will enable the community to establish datasets more easily for training and testing purposes (software and human). The inclusion of ontologized PHA4GE QC tags will also make datasets and quality control results FAIR (Findable, Accessible, Interoperable, and Reusable) [27].

## Theory and implementation

### Data needs assessment

The members of PHA4GE are involved in many sequencing initiatives and surveillance networks, and as a result, have a broad collective experience in developing solutions to microbial bioinformatics challenges. Requests for standardized quality control tags were made to PHA4GE from members of the wider public health and research communities via direct communication and social media. The range and types of common quality control issues were identified through a survey via member networks. The proposed list of quality control tags was circulated for feedback within the PHA4GE community and was improved based on feedback.

### Development of standardized quality control tags

After reviewing lower quality bacterial and viral datasets to determine the most common reasons for QC failure, the list of reasons and associated information was categorized and structured according to the fields and values in Table 1.

**Table 1.** Standardized fields and values for annotating quality control information in shared pathogen genomics datasets

Field	Definition	Ontology ID	Data type	Values	Example
Quality control method name	The name of the method used to assess whether a sequence passed a predetermined quality control threshold.	GENEPIO:0100557	String	No prescribed values	ncov-tools
Quality control method version	The version number of the method used to assess whether a sequence passed a predetermined quality control threshold.	GENEPIO:0100558	String	No prescribed values	1.2.3
Quality control determination	The determination of a quality control assessment.	GENEPIO:0100559	Enums	No quality control issues identified [GENEPIO:0100562]; sequence passed quality control [GENEPIO:0100563]; sequence failed quality control [GENEPIO:0100564]; minor quality control issues identified [GENEPIO:0100565]; sequence flagged for potential quality control issues [GENEPIO:0100566]; quality control not performed [GENEPIO:0100567]	Sequence failed quality control [GENEPIO:0100564]
Quality control issues	The reason contributing to, or causing, a low quality determination in a quality control assessment.	GENEPIO:0100560	Enums	Low quality sequence [GENEPIO:0100568]; sequence contaminated [GENEPIO:0100569]; low average genome coverage [GENEPIO:0100570]; low percent genome captured [GENEPIO:0100571]; read lengths shorter than expected [GENEPIO:0100572]; sequence amplification artefacts [GENEPIO:0100573]; low signal to noise ratio [GENEPIO:0100574]; low coverage of characteristic mutations [GENEPIO:0100575]; taxonomic designation inconsistent across methods [GENEPIO:0101038]	Low average genome coverage [GENEPIO:0100570]
Quality control details	The details surrounding a low quality determination in a quality control assessment.	GENEPIO:0100561	String	No prescribed values	CT value of 39. Low viral load. Low DNA concentration after amplification.

The QC attributes were then mapped to existing ontologies, and ontology terms were created for tags with no existing equivalent and made publicly available in the Genomic Epidemiology Ontology (GenEpiO, https://github.com/GenEpiO/genepio) in order to make the attributes FAIR (Findable, Accessible, Interoperable, Reusable). Ontology picklist term labels include ontology identifiers, which are presented in square brackets and include an ontology prefix (identifying the source ontology) and a numerical identifier (provided by the source ontology), e.g. ‘sequence failed quality control [GENEPIO:0100564]’. QC terms should always include their ontology identifiers, and while user-facing contextual data records in repositories and databases can present textual labels in interfaces, records in backend database tables/structures should ideally prioritize use of the identifiers over the accompanying textual label as the latter may vary across systems due to institutional preferences in terminology (as with the use of synonyms) whereas the identifiers represent the ‘ground truth’ of the standard.

Definitions were also developed, along with recommendations for their use in INSDC sequence submissions (in collaboration with INSDC representatives). The QC attributes were made publicly available on GitHub in October 2022, and included in a specially designed SRA submission form for pathogen sequence data (available on GitHub). GenEpiO QC terms can be searched using different ontology lookup services such as the EMBL-EBI OLS (https://www.ebi.ac.uk/ols/index), and downloaded as part of the GenEpiO web ontology language file available through the Open Biological and Biomedical Ontology (OBO) Foundry (https://obofoundry.org/ontology/genepio.html). The QC tags are also available as a bespoke JSON file through the PHA4GE QC GitHub repository, as well as field and term reference guides providing definitions and further guidance for usage.

As part of the testing and implementation process, QC contextual data tags were reviewed and evaluated by GenomeTrakr scientists for tagging known quality control issues in wastewater metagenomics datasets used for SARS-CoV-2 surveillance. The fields were added to the prescribed GenomeTrakr submission requirements, along with additional values for GenomeTrakr-specific pipelines and analyses.

### Best practices for use

Quality control of genomic sequences is a set of critical bioinformatics processes that are used to characterize quality properties of the data. While there are common processes that are often applied to datasets (e.g. assessment of read quality, coverage, GC content, potential contamination), quality control can be highly variable in terms of the methods and software used, the metrics and thresholds used as criteria for assessment, and the use cases for which the data is being evaluated. Not all QC metrics are considered appropriate for all uses of the data, and some use cases can be very specific for different public health priorities.

Often, public health surveillance programmes use genomics for specific applications which may have strict QC thresholds. If generated data does not meet those thresholds, labs may not feel like it is appropriate (or may not have permission) to share data that does not meet those strict requirements. However, it is important to recognize that data that is considered insufficient for one purpose may be appropriate and usable for other public health applications. For example, a SARS-CoV-2 consensus assembly with a failed quality status for low percent genome coverage may not be suitable to include in a phylogeny; however, that same assembly may be suitable for other applications if the Spike gene has full coverage. A bacterial assembly for a healthcare-associated pathogen may have low mean read depth across the genome with a high contig number, making it unsuitable for genome-wide SNP comparisons for outbreak clustering; however, key antimicrobial resistance genes may have been captured in full. In these cases, the data is still suitable for particular applications and it would benefit the public health community to submit this data to public repositories. This can also improve representation of valuable genomic data from under-represented countries, where genomic sequencing output may be distributed across a larger number of samples to reduce costs.

The use of QC tags in contextual data records helps to communicate how datasets have been evaluated so that data users can understand the methods and parameters previously applied, to better evaluate the uses of the data for their own purposes. It should also be recognized that public repositories like the INSDC accept data of varying quality and will accept QC tags as a means of making different kinds of datasets more discoverable.

As such, users of QC tags should not place too much weight on ‘pass/fail’ results which can be context dependent, but rather on the methods and issues provided by the tags. Also, it is very possible that a certain method may not yield a pass/fail result, but rather provide warnings and flags that help inform data users rather than indicate strict and universal acceptance or rejection. Also, owing to the differences in software algorithms, QC criteria, flags and reported issues, QC outputs and results may not match values in QC tag picklists. As such, converting QC results into QC tags will likely require interpretation on the part of the data provider. There are usually no ‘right’ answers when applying QC tags – the tags that make the most sense for a given scenario are up to the discretion of the data provider. Furthermore, while PHA4GE has attempted to provide tags to address common issues, the picklists are not exhaustive. PHA4GE looks to the community to help co-create resources. If users feel certain tags are missing, they are urged to communicate new data needs either via email (datastructures@pha4ge.org) or via GitHub using the New Term Request template (see instructions on GitHub).

Below are a few simple recommendations for implementing the QC tags (also available in the Field Reference Guide available at GitHub).

1. Providing the name of the method used for quality control is very important for interpreting the rest of the QC information. A method name should always be included (do not include additional QC tags if no method name is provided).
2. Method names can be provided in the form of a name of a pipeline or a link to a GitHub repository. Multiple methods should be listed and separated by a semicolon.
3. Methods updates can make big differences to their outputs. The version of the method used for quality control should be included.
4. The method version can be expressed using whatever convention the developer implements (e.g. date, semantic versioning). If dates are used for version control, the date should be provided in ISO 8601 format ‘YYYY-MM-DD’. If semantic versioning is used for version control, the semantic label should be provided as X.Y.Z where X is the major version, Y is the minor version, and Z is the patch version (see https://semver.org/ for more details).
5. Multiple methods can be included, separated using a semicolon.
6. If multiple methods were used, record the version numbers in the same order as the method names. Separate the version numbers using a semicolon.
7. Multiple ‘quality control determination’ and ‘quality control issues’ values can be included as appropriate, separated using a semicolon.
8. If a pick list does not contain a desired value, a new term request should be submitted to PHA4GE via the QC Tag GitHub repository issuetracker New Term Request form (described below under ‘Community Development and Maintenance’).

### Worked examples

To illustrate the many real-world applications of QC tags, we have compiled a set of different scenarios and worked examples below. In doing so, we hope the examples ‘give back power to the data’ and users are empowered to share a greater variety of data.

Tool names and version numbers are underlined in the scenarios.

A list of method names used in the worked examples is summarized in Table 2 below.

**Table 2.** Summary of the quality control assessment software and methods used in the worked examples

Quality control method name
FastANI
FastQC
Kraken2
Mash
Nextclade
ncov-tools
PHoeNIx
Quast
samtools depth
Samtools
VADR

Worked examples:

1. Ncov-tools 1.9.1 produces an ‘excess ambiguity’ flag based on the presence of >5 ambiguous sites in the consensus sequence, which may be indicative of contamination or a real mixed infection (no pass/fail provided). This example highlights how to interpret issues with no pass/fail indications.

*quality controlmethod name*: ncov-tools

*quality controlmethod version*: 1.9.1

*quality controldetermination*: sequence flagged for potential quality control issues [GENEPIO:0100566]

*quality controlissues*: sequence contaminated [GENEPIO:0100569]

*quality controldetails*: >5 ambiguous sites in consensus, sequence indicative of contamination or real mixed infection

2. Ncov-tools 1.9.1 detects a number of unexpected frameshift mutations, but neither calls a pass or fail result. This example highlights how to interpret issues with no pass/fail indications.

*quality controlmethod name*: ncov-tools

*quality controlmethod version*: 1.9.1

*quality controldetermination*: sequence flagged for potential quality control issues [GENEPIO:0100566]

*quality controlissues*: excess frameshift mutations detected [GENEPIO:0100751]

*quality controldetails*: Not Applicable [GENEPIO:0001619]

3. Samtools depth 1.19 outputs low read depth at positions which confer antimicrobial resistance in a *Mycobacterium tuberculosis* assembly, which prevents identification of a potential resistance profile in this specimen. This example highlights how to tag sequences with lower than expected/required characteristic mutations.

*quality controlmethod name*: samtools depth

*quality controlmethod version*: 1.19

*quality controldetermination*: sequence failed quality control [GENEPIO:0100564]

*quality controlissues*: low coverage of characteristic mutations [GENEPIO:0100575]

*quality controldetails*: low DNA concentration

4. FastQC 0.12.0 produces a report with low per base sequence quality, two peaks for per sequence GC content, and an average GC content that is higher than expected. This example highlights how to tag suspected contamination detected by GC content issues.

*quality controlmethod name*: FastQC

*quality controlmethod version*: 0.12.0

*quality controldetermination*: sequence failed quality control [GENEPIO:0100564]

*quality controlissues*: low quality sequence [GENEPIO:0100568]; sequenced contaminated [GENEPIO:0100569]

*quality controldetails*: contaminated cultured isolate

5. Kraken2 2.1.3 produces a report with percentage of reads assigned to each taxon in the database with a considerable percentage of reads belonging to a different species/taxon than the one targeted, indicating contaminating host reads. This example highlights how to tag suspected contamination detected by high non-targeted species abundances detected in reads.

*quality controlmethod name*: Kraken2

*quality controlmethod version*: 2.1.3

*quality controldetermination*: sequence flagged for potential quality control issues [GENEPIO:0100566]

*quality controlissues*: low signal to noise ratio [GENEPIO:0100574]

*quality controldetails*: high host reads present from clinical specimen

6. Nextclade 3.1.0 provides a clade designation for an MPOX viral assembly and produces quality flags indicating low percent genome coverage and high number of Ns. This example highlights how to tag sequences with low coverage and base quality.

*quality controlmethod name*: Nextclade

*quality controlmethod version*: 3.1.0

*quality controldetermination*: sequence failed quality control [GENEPIO:0100564]

*quality controlissues*: low percent genome captured [GENEPIO:0100571]

*quality controldetails*: Ct value of 35

7. Quast 5.2.0 produces an assembly quality report for a bacterial genome assembly and indicates higher contig number than expected for a particular pathogen Samtools 1.19 coverage output indicates only 15× mean depth of coverage for the assembly, lower than the threshold to be included for downstream phylogenetic analysis. This example highlights how to tag processes that utilize the outputs of more than one tool.

*quality controlmethod name*: Quast, Samtools

*quality controlmethod version*: 5.2.0, 1.19

*quality controldetermination*: minor quality control issues identified [GENEPIO:0100565]

*quality controlissues*: low quality sequence [GENEPIO:0100568]; low average genome coverage [GENEPIO:0100570]

*quality controldetails*: high number of samples multiplexed on same sequencing run

8. Mash 2.3, Kraken2 2.1.3, and FastANI 1.34 provide conflicting taxonomic classification results for a bacterial culture that was submitted to the laboratory to determine species identification. The reports generated produce inconsistent results compared with the taxonomic identification assigned by standard microbiological methods. The sequenced genome has otherwise passed all other quality metrics and contamination, or a sample mix-up, has been ruled out. This example highlights how to tag QC processes that integrate *in silico* and wet-lab information and for data from pathogens that may not be well-represented in public repository databases.

*quality controlmethod name*: Mash; Kraken2; FastANI

*quality controlmethod version*: 2.3; 2.1.3; 1.34

*quality controldetermination*: sequence flagged for potential quality control issues [GENEPIO:0100566]

*quality controlissues*: taxonomic designation inconsistent across methods [GENEPIO:0101038]

*quality controldetails*: WGS species identification inconsistent across multiple taxonomic classification methods and inconsistent with identification from culture. Potential novel species/subspecies and/or underrepresented in public repository databases.

9. NCBI’s Viral Annotation DefineR (VADR 1.6.3) generates alerts for a viral assembly, including early stop codon, frameshift mutation, and low feature similarity. This example highlights how to tag multiple detected issues.

*quality controlmethod name*: VADR

*quality controlmethod version*: 1.6.3

*quality controldetermination*: sequence flagged for potential quality control issues [GENEPIO:0100566]

*quality controlissues*: low percent genome captured [GENEPIO:0100571]; low coverage of characteristic mutations [GENEPIO:0100575]; excess frameshift mutations detected [GENEPIO:0100751]

*quality controldetails*: multi-freeze thaw cycles for specimen. Amplicon drop-out.

10. The US CDC’s PHoeNIx 2.1.0 pipeline, a short-read pipeline for healthcare-associated and antimicrobial resistant pathogens, produced an output report with an overall ‘SUCCESS’ status, indicating all 27 quality metrics passed as ‘SUCCESS’. This example highlights how to tag a sequence that passed quality control metrics using multiple tools incorporated into a workflow.

*quality controlmethod name*: PHoeNIx

*quality controlmethod version*: 2.1.0

*quality controldetermination*: sequence passed quality control [GENEPIO:0100563]

*quality controlissues*: Not Applicable [GENEPIO:0001619]

*quality controldetails*: Not Applicable [GENEPIO:0001619]

Synthetic data

QC tags can still be applied in the special case of synthetic data (artificially created rather than generated by sequencing public health samples), or sequence data generated from lab constructs (such as artificially spiked samples). However, these datasets *must not* inherit any sample metadata from any original biological sources and should be marked as artificial using the field ‘experimental specimen role type’ and the tag ‘Synthetic lab construct [GENEPIO:0101039]’. Details regarding the synthesis of the data can be provided in the free text ‘experimental specimen details’ field. Further guidance on tagging synthetic data will be discussed elsewhere (manuscript in preparation).

11. A lab deliberately mixed different SARS-CoV-2 variants at various concentrations to simulate contamination or mixed infections. They then sequenced the simulated samples to determine the lower limits of detection of each variant. Analysis of the synthetic data provided insights into the ability of the protocol to identify mixed infections, detect contamination, and to determine the extent that it might interfere with accurate variant identification. This example highlights how QC tags can be used in conjunction with other fields to capture methods optimization information and share synthetic data and lab constructs.

*quality controlmethod name*: ncov-tools

*quality controlmethod version*: 1.9.1

*quality controldetermination*: sequence passed quality control [GENEPIO:0100563]; sequence failed quality control [GENEPIO:0100564]

*quality controlissues*: sequence flagged for potential quality control issues [GENEPIO:0100566]; low coverage of characteristic mutations [GENEPIO:0100575]; sequence contaminated [GENEPIO:0100569]; low signal to noise ratio [GENEPIO:0100574]

*quality controldetails*: Various quality control issues may arise depending on the mixture ratio for each sample and the particular mutations of each variant. Where one sample was added at a low level the sequence may pass quality control.

*experimental specimen role type*: synthetic lab construct [GENEPIO:0101039]

*experimental specimen details*: two different SARS-CoV-2 variants were mixed at varying ratios and whole genome sequenced to determine if our QC analysis would flag them with quality issues and at what level a mixture or potential contamination would be detected.

### Real-world implementations of standardized quality control tags

PHA4GE has collaborated with the INSDC in the design and application of the QC tags. Both organisations recommend the inclusion of the QC tags in submissions, and that the attributes should be included as user-defined fields in SRA (NCBI; https://www.ncbi.nlm.nih.gov/sra) or included interactively or programmatically in experiment metadata via the ENA submission system (note: the Webin command line programme does not accept user-defined attributes) ([28]; https://www.ebi.ac.uk/ena/browser/home). To facilitate inclusion in NCBI submissions, PHA4GE has created a modified SRA submission form containing PHA4GE QC fields with drop-down menus of prescribed values which is available at https://github.com/pha4ge/contextual_data_QC_tags. Step-by-step submission instructions for SRA are available in Protocols.io at dx.doi.org/10.17504/protocols.io.rm7vzjyj5l×1/v1. Submission instructions for ENA can be adapted from PHA4GE’s SARS-CoV-2 Protocols.io protocol, which can be found at https://www.protocols.io/view/sop-for-populating-ebi-submission-templates-ena-3byl474wjlo5/v1.

The GenomeTrakr network conducted a pilot study utilizing PHA4GE QC tags, tailored specifically for targeted amplicon sequencing of SARS-CoV-2 in wastewater samples [26]. The pilot study involved 19 GenomeTrakr laboratories plus FDA-CFSAN laboratories (combination of federal and state laboratories). Given the well-defined quality control metrics and thresholds of the study, as well as guidance provided by the FDA, users found the QC tags relatively easy to apply. The customizations and usage examples of these QC tags are detailed in Table 3. Table 4 presents example SRA records featuring these attributes. This project yielded 2255 short read sequences, all tagged with PHA4GE QC attributes. Of these, 61 % were classified as having ‘no quality control issues’, 10 % had ‘minor quality control issues’, 28 % had ‘potential quality control issues’, and 1 % had ‘significant quality control issues’. Additionally, the ‘quality_control_method_name’ and ‘quality_control_method_version’ fields provide essential QC methodological details, adding valuable context to the dataset even when no specific issues are reported.

**Table 3.** Implementation and updates to the PHA4GE quality control attributes applied by GenomeTrakr network

Attribute name	Description	Guidance	Term lists
quality_control_method_name	Name of quality control pipeline, software, or method	Populate using a term from the picklist	GalaxyTrakr SSQuAWK;CFSAN Wastewater Analysis Pipeline (C-WAP)
quality_control_method_version	Version number		
quality_control_determination	User determined assessment of data quality	Populate using a term from the picklist	No quality control issues identified; minor quality control issues identified; sequence flagged for potential quality control issues; not performed
quality_control_issues	Quality control issues relevant for the project or data type	Populate using a term from the picklist	Low quality sequence; sequenced contaminated; low average genome coverage; low percent genome captured; read lengths shorter than expected; sequence amplification artefacts; low signal to noise ratio; low coverage of characteristic mutations
quality_control_details	Free text attribute capturing custom entry	Free text entry	None

It should be noted that GenomeTrakr also includes other PHA4GE contextual data fields for describing their methods in their SRA submissions. These attributes include amplicon_PCR_primer_scheme; amplicon_size; dehosting_method; and sequence_submitter_contact_email. These additional prescribed fields are outlined in the PHA4GE SARS-CoV-2 Contextual Data Specification (https://github.com/pha4ge/SARS-CoV-2-Contextual-Data-Specification).

**Table 4.** Example GenomeTrakr SRA records illustrating PHA4GE QC contextual data tag use

Data provider	Surveillance network	SRA accession	Run record
Washington State Department of Health	GenomeTrakr wastewater project	SRR21205381	SRR21205381 Run Record
US FDA, Centre for Food Safety and Applied Nutrition	GenomeTrakr wastewater project	SRR19851129	SRR19851129 Run Record
US FDA, Centre for Food Safety and Applied Nutrition	GenomeTrakr wastewater project	SRR20046849	SRR20046849 Run Record
New Jersey Department of Agriculture	GenomeTrakr wastewater project	SRR20428498	SRR20428498 Run Record
Texas Department of State Health Services	GenomeTrakr wastewater project	SRR20018633	SRR20018633 Run Record

PHA4GE Contextual Data QC Tags in SRA can be found in the Run records (the hyperlink that starts with SRR in the ‘Run’ chart near the bottom of the webpage). This information can be accessed by clicking the hyperlink and then clicking the ‘Show additional attributes’ link in the Run section.

### Annotation limitations and considerations

The QC tags are intended to address issues pertaining to different types of sequencing techniques (single isolate or targeted sequencing, metagenomics). Not all tags may apply to all techniques and so where they are not appropriate then they should not be used. The tags are also intended to describe QC results of sequence data rather than downstream analytical results (e.g. intended for raw reads, consensus sequences or assemblies, species identification rather than phylogenies). Owing to the wide variety of quality control software available, and the differences in criteria and thresholds, the application of these attribute tags may be subjective and dependent on the QC processes performed. To better evaluate and interpret the QC determinations proposed, it is recommended that other information pertaining to QC be included in other contextual data fields not specified in this work (i.e. choice of reference genome), and that the tags be interpreted considering the other methodological metadata included in the record (i.e. BioSample, Experiment/SRA contextual data). The controlled vocabulary attributes are intended for high-level triage purposes rather than capturing all methods in detail. However, information affecting the selection of one tag over another can also be included in the ‘quality_control_details’ field. It is also important to note that the quality control tags refer to a particular sample obtained at one point in time, and not the comparison of a set of samples across time or from different tissues of the same host.

### Community development and maintenance

While the initial list of standardized QC fields and values was developed by PHA4GE through community consultation, we recognize it will need to change over time. To ensure the list reflects current QC issues across pathogens and methods (e.g. sequencing techniques, bioinformatics analyses), a mechanism for requesting additional QC tags was created via the PHA4GE QC GitHub repository Issue tracker (New Term Request [NTR] form). A template for submitting new terms is available and community members are welcome to submit suggestions for new terms by following the instructions provided with the template. Suggestions will be evaluated and periodic updates to the list will be performed.

## Discussion

A major goal of pathogen genomics surveillance programmes is to produce high quality data for use in public health analyses and decision-making. Owing to time, personnel and resource limitations, samples that yield sequences of borderline or slightly poorer quality cannot often be re-sequenced. This sequence data, while perhaps not suitable for surveillance or outbreak analysis, is still useful for the development of tools, the optimization and validation of quality frameworks and sequencing processes, as well as for bioinformatics training purposes. Useful datasets for testing and training purposes can include sequences containing contamination, low yields and/or low average genome coverage, shorter than expected read lengths, sequence amplification artefacts, low signal-to-noise ratio, and low coverage of characteristic mutations.

Due to the lack of standardized attributes in contextual data records, purposefully identifying sub-optimal quality datasets in public repositories is difficult. The PHA4GE Contextual Data QC Tag Specification provides a set of five fields which can be included as user-defined contextual data in public repository raw read sequence submissions. While PHA4GE encourages the use of these fields and terms in any repository, not all public repositories have the mandate or the ability to include user-defined attributes. The PHA4GE tags are implementable in submissions to the INSDC (in SRA [NCBI, DDBJ] and as ‘Experiment’ contextual data in ENA). The tags have been used by the GenomeTrakr pathogen surveillance network to flag general quality control issues (or the lack thereof), as well as to provide additional quality control methods information.

The GenomeTrakr implementation demonstrates how the generic PHA4GE tags can be customized according to initiative-specific needs. GenomeTrakr adds standardized names of QC pipelines used by different data providers in the ‘quality_control_method_name’ field, and has created other ‘quality_control_issues’ tags that were subsequently added to the PHA4GE prescribed list, i.e. ‘low coverage of characteristic mutations’. We anticipate that as the tags are implemented for different organisms and initiatives, there may be other useful tags that should be included in the PHA4GE list. PHA4GE encourages feedback and suggestions from the community via the New Term Request form on GitHub. By sharing community needs and requests with PHA4GE in this way, we are able to work with ontology developers and public repository scientists to make new standardized vocabulary available through different channels. Also, it is possible to create different collections of specifications so that tags are honed for particular use cases. It should be noted that the free-text ‘quality control details’ field enables data generators to add notes on their QC assessments that do not fit into the other standardized fields. While free text is often less useful than controlled vocabulary, community feedback on the tags suggested a degree of flexibility would be beneficial to capture the broad scope of possible QC information and to accommodate unexpected results, which could be missed if controlled vocabulary is too rigid. As data is shared and information is propagated, PHA4GE also recommends updating all private and public records (e.g. SRA/ENA) with known quality control issues if detected at a later date.

There are many elements to standardizing quality control including specifying types of metrics and their parameters and thresholds, selecting and documenting tools and algorithms, prescribing different checkpoints in wet- and dry-lab processes, and so on. However, the PHA4GE Contextual Data QC Tag Specification does not delve into these more in-depth aspects, but rather the attributes act as quick, searchable, downstream flags for overall outcomes of QC assessments. Further development in standardized QC language and harmonized QC threshold for such nuanced aspects of QC frameworks is therefore needed. PHA4GE is currently undertaking a study to understand the breadth of quality control methods, software, metrics and thresholds for different pathogens and sequencing applications so that such standardized QC language can be developed in the future. Presently, we hope that these simple tags will help improve communication around quality control in public repositories, as well as make datasets of variable quality easier to identify.

## Abbreviations

DDBJ: DNA Data Bank of Japan
EMBL-EBI: European Molecular Biology Laboratory European Bioinformatics Institute
ENA: European Nucleotide Archive
FAIR: Findable, Accessible, Interoperable, and Reusable
FDA: US Food & Drug Administration
GenEpiO: Genomic Epidemiology Ontology
INSDC: International Nucleotide Sequence Data Collaboration
ISO: International Organization for Standardization
NCBI: National Center for Biotechnology Information
NTR: new term request
OBO: open biological and biomedical ontology
OLS: ontology look-up service
PHA4GE: public health alliance for genomic epidemiology
QC: quality control
SARS-CoV-2: severe acute respiratory syndrome coronavirus 2
SRA: sequence read archive
